# MAPbBr_3_ perovskite solar cells *via* a two-step deposition process

**DOI:** 10.1039/c9ra02036e

**Published:** 2019-04-26

**Authors:** Hanadi Mehdi, Asya Mhamdi, Riadh Hannachi, Abdelaziz Bouazizi

**Affiliations:** Équipe Dispositifs Électroniques Organiques et Photovoltaïque Moléculaire, Laboratoire de La Matière Condensée et des Nanosciences, Faculté des Sciences de Monastir, Université de Monastir. Avenue de L'environnement 5019 Monastir Tunisia hanadi.mehdi92@gmail.com mhamdiasya@gmail.com; Laboratoire des Energies et des Matériaux (LabEM), Institut Supérieur d’Informatique et des Techniques de Communication, Université de Sousse Hammam Sousse 4011 Tunisia

## Abstract

Organometal halide perovskite solar cells are becoming one of the most competitive emerging technologies. They have reached a power conversion efficiency (PCE) of 22.7% in 10 years. Their high efficiency and simple fabrication process render perovskite solar cells a promising player in the field of third-generation photovoltaics. The deposition methods play an important role in the fabrication of a high quality films. In this paper, we report the preparation of methylammonium lead bromide (MAPbBr_3_) thin film using a two-step method based on the transformation of PbBr_2_ into MAPbBr_3_ perovskite after dipping in a MABr solution. The effects of the dipping time and the annealing time on the photovoltaic, optical and structural properties of the devices were studied. The dipping time treatments of the inorganic film in organic solution were conducted from 30 s to 15 min. The obtained result showed that the PCE of the devices was improved with the increase of dipping time. In addition, an increase of annealing time induces an enhancement of the perovskite properties. Furthermore, the as-fabricated perovskite solar cell dipped and annealed for 10 min exhibited the highest power conversion efficiency of 4.8% with a short circuit current density of 16.16 mA cm^−2^, an open circuit voltage of 0.84 V, and a fill factor of 35.50.

## Abstract 2

Organometal halide perovskite solar cells are becoming one of the most competitive emerging technologies.

## Graphical abstract

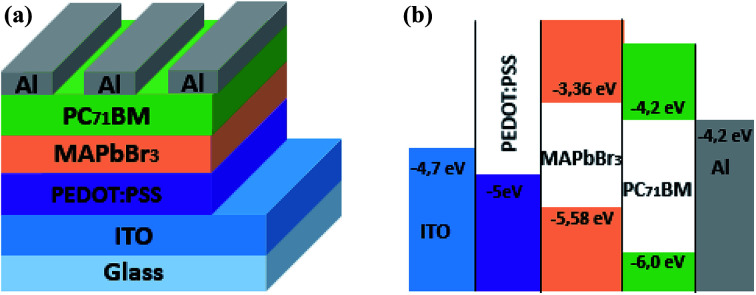

## 1. Introduction

Recently, hybrid organic–inorganic perovskite materials, such as CH_3_NH_3_PbX_3_ (X = Cl, Br, I), have attracted increasing attention in the field of solar cell applications due to their inherent properties.^1–4^ These new materials ignite powders in the world of photovoltaic cells. Their power conversion efficiency (PCE) has recently achieved 23% under 1 sun condition (100 mW cm^−2^ AM 1.5G)^5^ which is the consequence of their excellent advantages. These advantages including low cost, facile preparation process, good absorption of sunlight with a broad spectrum of absorption covering the visible and near infrared spectra,^6,7^ high extinction coefficient,^8^ small exciton binding energy,^9,10^ and excellent mobility of the charge carriers make it possible to have a long diffusion length, which is greater than 1 μm.^11,12^

The first incorporation of organic–inorganic hybrid perovskite materials was in a mesoscopic dye-sensitized solar cell (DSSC) reported by Michael Gratzel in 2009.^13^ However, the manufacture of this type of cells requires a processing temperature up to 500 °C to sinter the TiO_2_ support, which renders them incompatible with flexible substrates. After that, a planar perovskite hybrid solar cell has been developed.^14–17^

Generally, the configuration of PSCs can be divided into two structures: the planar standard solar cells with the structure fluorine-doped tin oxide/ETL/perovskite/HTL/anode and the inverted structure, which is composed of ITO/HTL/perovskite layer/ETL/cathode. The most known HTL used the inverted is the PEDOT:PSS, whereas in the standard structure are PTAA and spiro-OMeTAD. The ETL materials used in the inverted structure are PCBM and C60, whereas in the conventional structure are TiO_2_, Al_2_O_3_, and ZnO, whereas the ETL.

The majority of the hybrid perovskite application was in planar standard structure.^18–20^ The first application of organic–inorganic perovskite materials in inverted solar cells was in 2013 by Jeng *et al.*^21^ which the perovskite was used as a donor and PC_61_BM as an acceptor to construct a perovskite/fullerene planar heterojunction hybrid solar cell. After that, several researchers have used the same structures.^22–25^ Several deposition methods were used in order to fabricate perovskite films, such as one step spin coating by mixing the two precursors in the same solvent. Which is widely used coating method to form a perovskite thin film on top of the mesoporous ETL,^26,27^ due to the lack of suitable solvents that can dissolve both components, and the high reaction rate of the perovskite component. This process often results in thin films with pinhole formation and incomplete surface coverage, which deteriorates the film quality and hampers the device performance.^28^ Other deposition methods have been used to coat the perovskite film, such as vapor-assisted solution process,^29^ Two step coating process,^30^ dual source co-evaporation,^31^ vapor deposition,^32^ spray and blade coating which are used for large area device fabrication.^33,34^

**Scheme 1:**
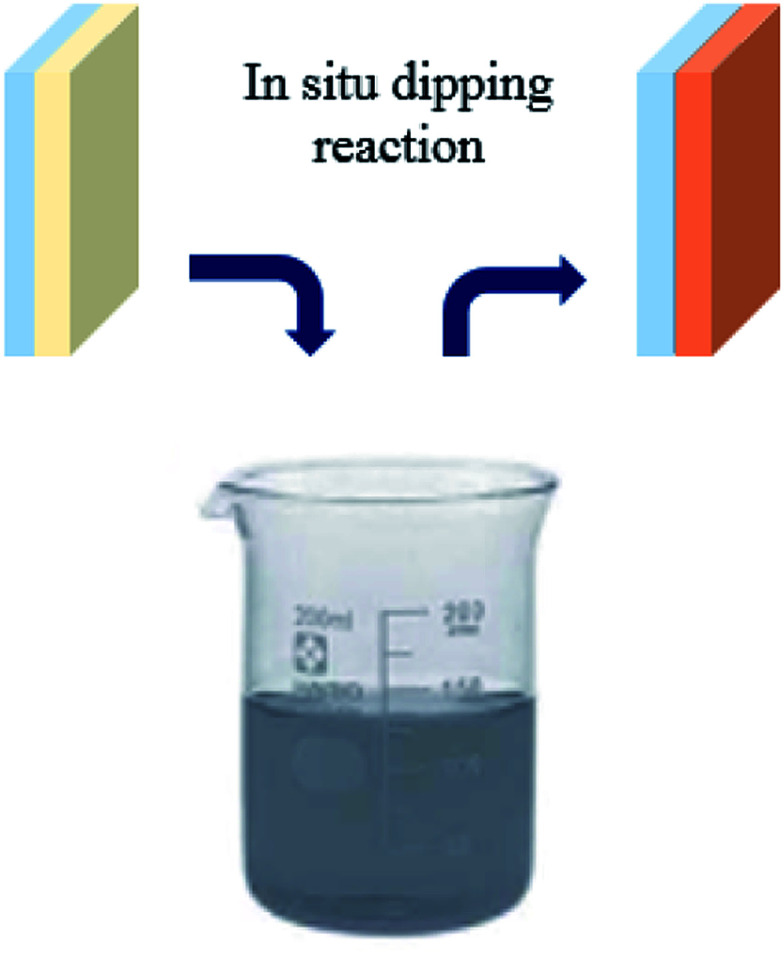
Sequential deposition method; dipping the PbBr_2_ film into MABr solution.

In this manuscript, we report a method of forming continuous, compact bromide perovskite MAPbBr_3_, (MA = CH_3_NH_3_), films by dipping the PEDOT:PSS/PbBr_2_ film into a solution of MABr in 2-propanol, to induce a solid liquid reaction. The key step is film growth *via in situ* reaction of the as-deposited film of PbBr_2_ with MABr solution (Scheme 1). In order to evaluate the effect of the dip-coating and annealing treatment we prepared different films with different dipping time (30 s, 1 min, 5 min, 10 min and 15 min) and different annealing time (1, 5 and 10 min).

Inverted planar perovskite solar cells with the structure of ITO/PEDOT:PSS/MAPbBr_3_/PC_71_BM/Al was fabricated and characterized using optical, structural and electrical tools.

## 2. Experimental section

### 2.1. Materials and methods

MABr and PbBr_2_ were purchased from Ossila, with a weight average molecular weight, respectively, (*M*_w_) = 111.96 g mol^−1^ and 367.01 g mol^−1^. The MAPbBr_3_ was used as absorber material and electron donating material while a soluble fullerene derivative, 6,6-phenyl C71-butyric acid methyl ester (purchased from Ossila Ltd.), was used as an acceptor material.

**Fig. 1:**
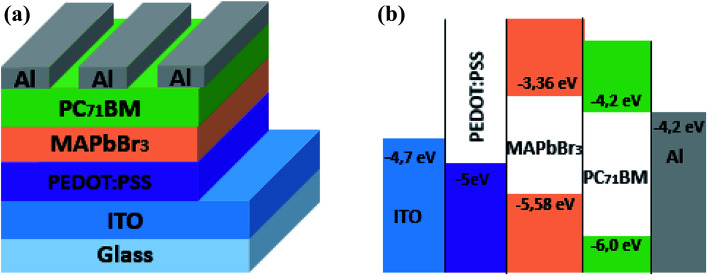
(a) Device structure of a MAPbBr_3_/PC_71_BM bilayer solar cell. (b) Schematic energy level diagram of ITO, PEDOT:PSS, MAPbBr_3_, PC_71_BM and Al.

A poly (3,4-ethylenedioxythiophene):poly (styrene sulfonate) (purchased from Ossila Ltd.) was used as hole transporting layer. The schematic and energy band diagrams of elaborated hybrid perovskite solar cells are shown in Fig. 1.

The inverted PSCs: ITO/PEDOT:PSS/MAPbBr_3_/PC_71_BM/AL was elaborated according to the following procedure:

The ITO substrates (Ossila Ltd.) were cleaned in sequential ultrasonic baths of desionized water, acetone, and isopropanol for 15 min, and subsequently dried in N_2_ gas flow. The PEDOT:PSS hole transporting layers was subsequently deposited onto the treated ITO substrate and annealed at 140 °C for 20 min. A solution of PbBr_2_ (367 mg ml^−1^) was dissolved in *N*,*N*-diméthylformamide (DMF), heat up to 60 °C and continuously stirred for 30 min, then deposited on top of the PEDOT:PSS layer by spin coating for 20 s at 6000 rpm. The obtained layers were annealed in vacuum at 100 °C for 10 min. Then they were dipping in a solution of CH_3_NH_3_Br dissolved in 2-propanol (10 mg/1 ml) for different dipping time and then annealed in vacuum at 100 °C for 10 min. A solution of PC_71_BM (10 mg ml^−1^) dissolved in 1,2-chlorobenzene (CB), was deposited on top of the prepared layer by spin coating at 1000 rpm for 45 s. The obtained films were then dried for 10 min at 100 °C. To complete the solar cell devices, a metal top electrode (Al) was deposited by thermal evaporation in high vacuum.

### 2.2. Instrumentation

The UV-vis spectra were performed using a Perkin Elmer Lambda 35 spectrophotometer. Photoluminescence spectra have been performed with a “Jobin Yvon-Spex Spectrum One” CCD detector, cooled at liquid nitrogen temperature. The X-ray diffraction (XRD) patterns of films were measured by A Siemens D5000 diffractometer. The current–voltage characteristics under illumination with Xe Oriel solar simulator were obtained with a Keithley 6430 source.

## 3. Results and discussion

### 3.1. Effect of dipping time

The transformation of PbBr_2_ layer into MAPbBr_3_ by dipping the samples in a solution of MABr is produced by the incorporation of PbBr_2_ films into a solution of MABr with a concentration of 10 mg mL^−1^. The effect of the reaction time on the properties of the MAPbBr_3_ films has been analyzed by the characterization of the obtained films after different dipping times: 30 s, 1 min, 5 min, 10 min and 15 min.

#### 3.1.1. XRD measurements

To study the variation of structural properties in MAPbBr_3_, we measured the X-ray diffraction of a series of samples with different dipping time of PbBr_2_ layers in the solution of MABr.

**Fig. 2:**
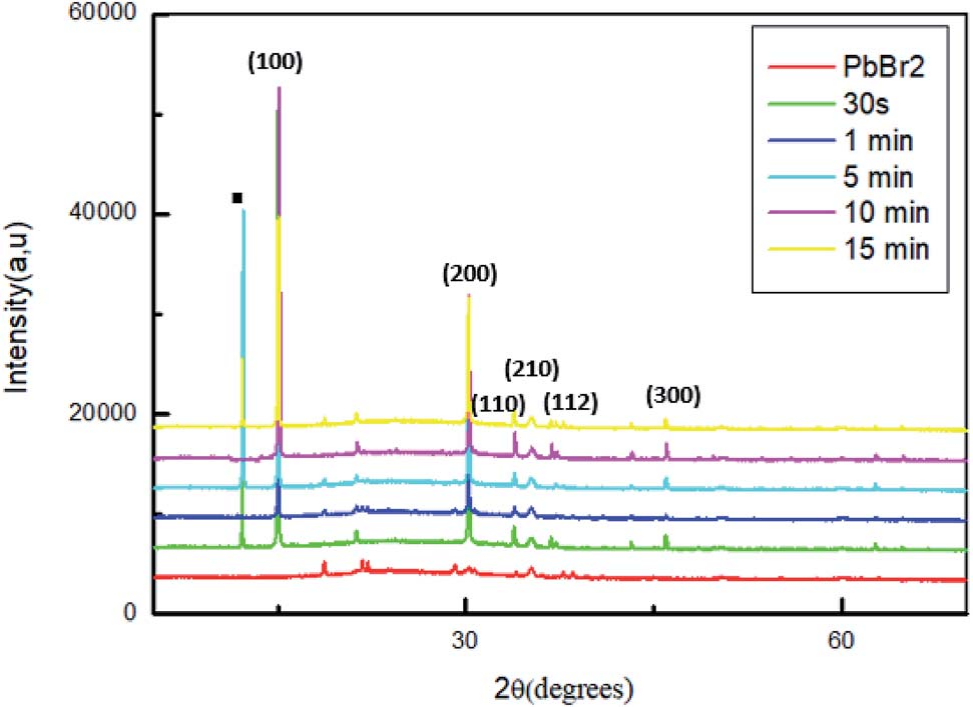
XRD patterns of MAPbBr_3_ films with different dipping time.

Fig. 2 shows the XRD patterns of the original PbBr_2_ films and of the same films exposed to MABr solution with different dipping times.

For all the sample, two strong diffraction peaks are located at 15.01° and 30.6° corresponding to the (100) and (200) planes respectively and other low peaks at 21.26°; 33.86°; 35.19° and 45.96 assigned to (110), (210), (112) and (300) respectively, which confirm the formation of a cubic perovskite structure.^35^

However, the conversion of the perovskite on exposure to the MAPbBr_3_ solution is incomplete, some sample contain the startly materials (PbBr_2_, MABr).^36^ Specially, for the films dipped at 30 s, 5 min and 15 min a strong diffraction peak indicated by a square symbol at 12.5°, which peak is attributed to the organic compound MABr.^26,37^

The higher intensity for the film dipped for 10 min may be attributed to a complete crystallization compared to the other films. Furthermore, at this stage of dipping time there are no additional peaks and the MABr was identified from XRD patterns, this behavior indicates a good crystallinity of the film.

#### 3.1.2. Optical band gap and photoluminescence

Changing the dipping time affect also the optical properties of the MAPbBr_3_ films. To investigate the effect of dip-coating method on the crystal structure of the perovskite film, the UV-vis absorption spectra of the CH_3_NH_3_PbBr_3_ perovskite film prepared from different dipping time were measured.

**Fig. 3:**
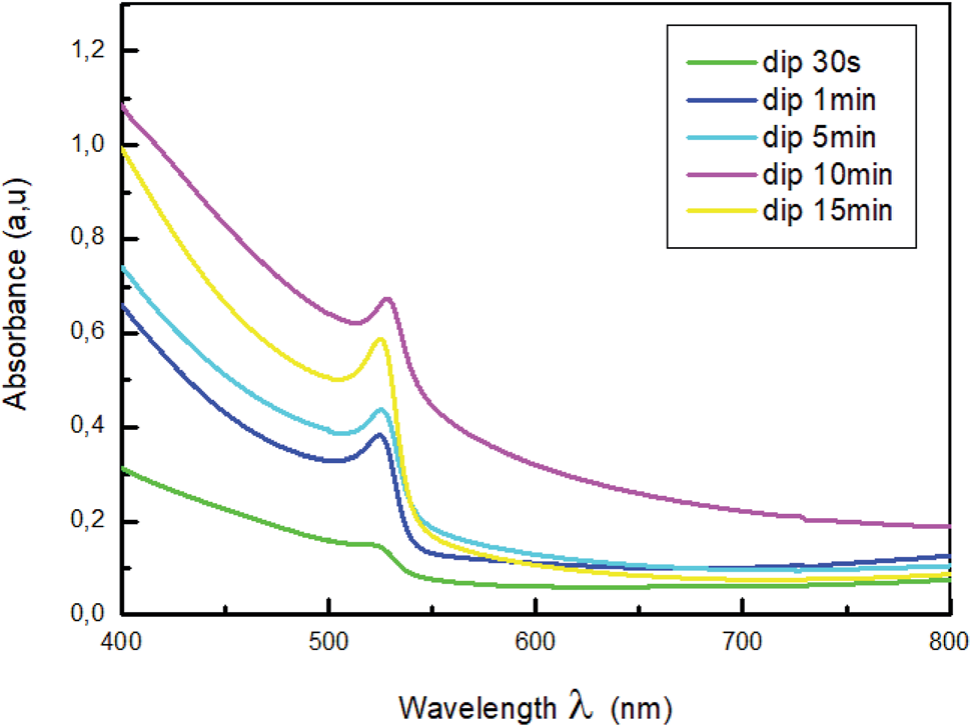
Absorption spectra of MAPbBr_3_ with different dipping time.

The normalized absorption spectra of MAPbBr_3_ films are displayed in Fig. 3. As shown in this figure, all the samples exhibit a characteristic absorption band around 528 nm corresponding to the material optical band gap *E*_g_ = 2.3 eV,^38,39^, with a range broad absorption of light ranging from visible to near-infrared region, which indicate the formation of MAPbBr_3_ perovskite on the substrate. Also, it is a sign of self-organization of the perovskite.^40^ The absorbance intensity increase and then decrease when the dipping time increased further, which indicate the change of crystallinity with the increase of the dipping time.

The higher optical density was obtained for the film dipped for 10; this behavior is due to the improvement of the crystallinity of the hybrid perovskite material.^41,42^

Therefore, the photoluminescence (PL) emission spectra of the perovskite layers were measured at room temperature.

**Fig. 4:**
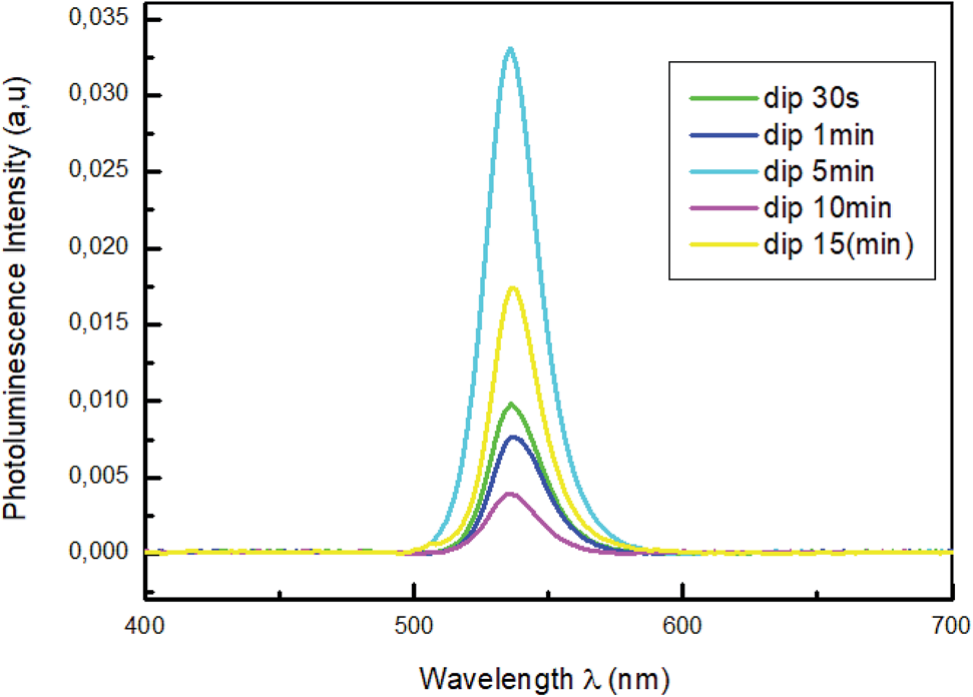
Photoluminescence spectra of MAPbBr_3_ with different dipping time.

Fig. 4 shows the photoluminescence spectra of MAPbBr_3_ thin films with different dipping times (30 s; 1 min; 5 min; 10 min and 15 min), in the 400–700 nm wavelength range and under an excitation at 375 nm to predominantly excite the perovskite film.

For all the samples a PL emission peaks centered in 535 nm were observed which is due to the exciton emission. The sample emission peaks show a slight shift towards the longest wavelength relative to the peak of the absorptions, which is known as the Stokes shift.^43^ The variation of absorption peak positions of these films does not induce a change in peak emission position of PL. The photoluminescence (PL) measurements in Fig. 4 reveal that the increase of the dipping time leads to strong quenching of the perovskite PL intensity. The reduced of PL intensity indicate a good dissociation of charges and a fast transport of holes from perovskite films to PEDOT:PSS layers which improve the film quality of MAPbBr_3_.^44^

#### 3.1.3. Photovoltaic performance

**Fig. 5:**
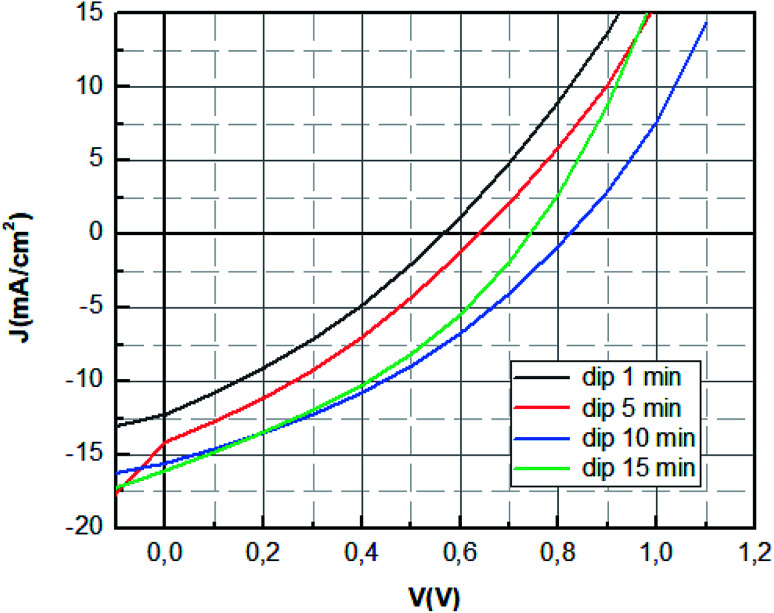
*J*–*V* characteristics of MAPbBr_3_ solar cell with different dipping time.

The *J*–*V* characteristics of hybrid perovskite solar cells with different dipping time are shown in Fig. 5. The average photovoltaic parameters, including short circuit current density (*J*_sc_), open circuit voltage (*V*_oc_), fill factor (FF), series resistance (*R*_s_) and PCE, are listed in Table 1.

**Table 1:** Photovoltaic parameters of ITO/PEDOT:PSS/MAPbBr_3_/PCBM/Al devices with different dipping time

Dip time (min)	*V*_oc_ (V)	*J*_sc_ (mA cm^−2^)	FF (%)	PCE (%)	*R*_s_ (ohm cm^2^)
1	0.57	14.69	29.64	2.47	11.04
5	0.64	15.12	30.90	2.99	7.42
10	0.84	16.16	35.50	4.85	6.87
15	0.74	16.55	34.91	4.29	5.66

The optimum photovoltaic performance is achieved for dipping time of 10 min, which displays a *J*_sc_ of 16.16 mA cm^−2^, a *V*_oc_ of 842 mV and FF of 35.50%. The device performances of MAPbBr_3_ perovskite solar cells were improved with the increase of the dipping time (1 min; 5 min; 10 min) as shown in Fig. 5. The increase of *V*_oc_ value can be mainly attributed to the enhancement of charge extraction. This variation is correlated with the extinction of the photoluminescence response, where the *V*_oc_ tension reaches its maximum value for the same dipping time, corresponding to the minimum of photoluminescence intensity. The improvement of the crystallinity of the layer causes the increase of the interface and later more interfacial area is present to extract the charge which reduces charge carrier losses.^45^ The enhancement is mainly also due to the increase of short circuit current, which was due to the enlarged contact area of the perovskite material with the PEDOT:PSS layer and the elimination of the recombination pathways owing to a film of perovskite with good crystallinity.^46^

However, the increase of the dipping time (15 min) leads to the decrease of the fill factor and the open circuit voltage. This can be explained by the decomposition of materials at a high dipping time into the starting product, which causes the increase of default or the decrease of charge transfer.

In this part, we have optimized the dip coating method. We have shown that the dip-coating time must be well determined in order to avoid the decomposition of the perovskite film. The deposit method plays an important role in the fabrication of a good quality perovskite film, but without a well-determined thermal annealing, this step will not have realized. Furthermore, one of the main parameters controlling the properties of the perovskite is the thermal annealing process, which is an essential step to initiate or accelerate the reaction between the organic and inorganic materials. It allows the improvement of the efficiency of the elaborated photovoltaic cells up to a certain limit. So determining the proper annealing temperature and the time of the annealing is an important step in optimizing the efficiency of the photovoltaic cells. The optimum temperature for the formation of a perovskite MAPbBr_3_ film has been much studied by the researchers, it was set at 100 °C.^47^ Our objects in the next part will not the determination of the proper annealing temperature but rather the annealing time needed to have good quality MAPbBr_3_ perovskite film. In this part, we present a study of the effect of the thermal annealing time on the structural and optical properties of MAPbBr_3_ films, deposited by the dip-coating method.

### 3.2. Effect of annealing temperature

#### 3.2.1. XRD measurements

**Fig. 6:**
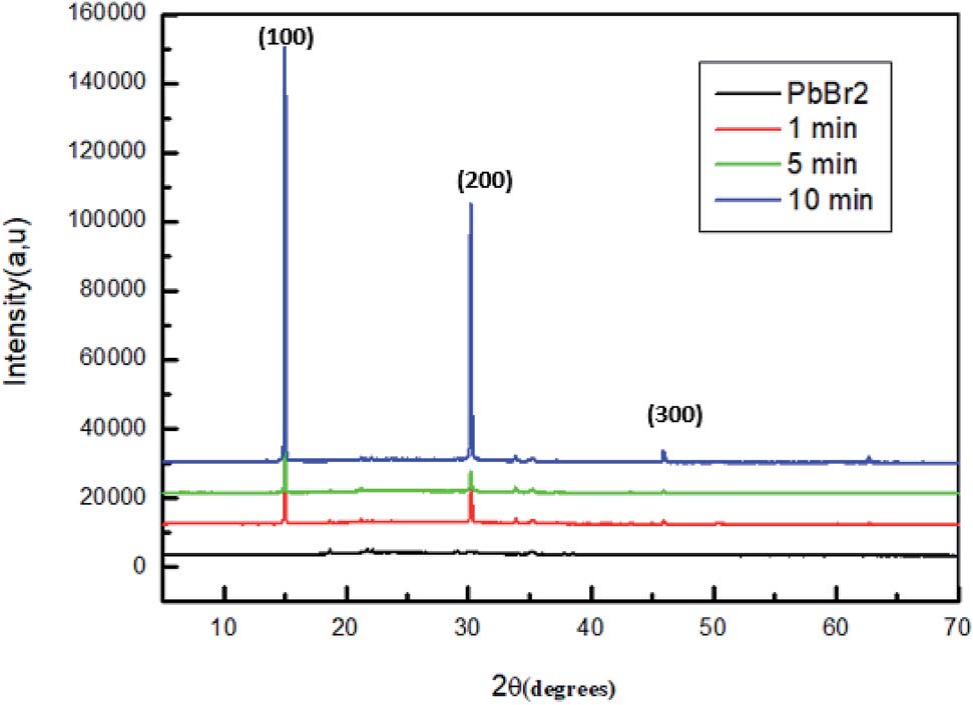
X-ray diffraction patterns of MAPbBr_3_ films annealed for different time.

The phase purity and the crystallinity of the films were confirmed with X-ray diffraction (XRD). Fig. 6 shows the XRD patterns of MAPbBr_3_ perovskite thin film deposited on the top of ITO/PEDOT:PSS substrate and heated at 100 °C for different annealing time. All the sample indicate the presence of diffraction peaks located at 15°, 30° and 45° corresponding to the (100), (200) and (300) planes, which confirms the formation of a cubic perovskite structure.

The intensity of the peaks located at 2*θ* = 15° and 30° increase gradually after the increase of the annealing time temperature up to *t* = 10 min. These behaviors indicate that the crystallinity of perovskite material is increased simultaneously.^48,49^

Compared with the film, which is annealed for 1 and 5 min, the conversion of PbBr_2_ into MAPbBr_3_ is not complete, which is well observed in the intensity of the peaks corresponding to (100) and (200) planes which is lower than that of the film annealed for 10 minutes.

#### 3.2.2. Optical measurements

To elucidate the effect of annealing temperature time in the elaborated devices, we have measured the absorbance spectra of the MAPbBr_3_ perovskite thin films.

**Fig. 7:**
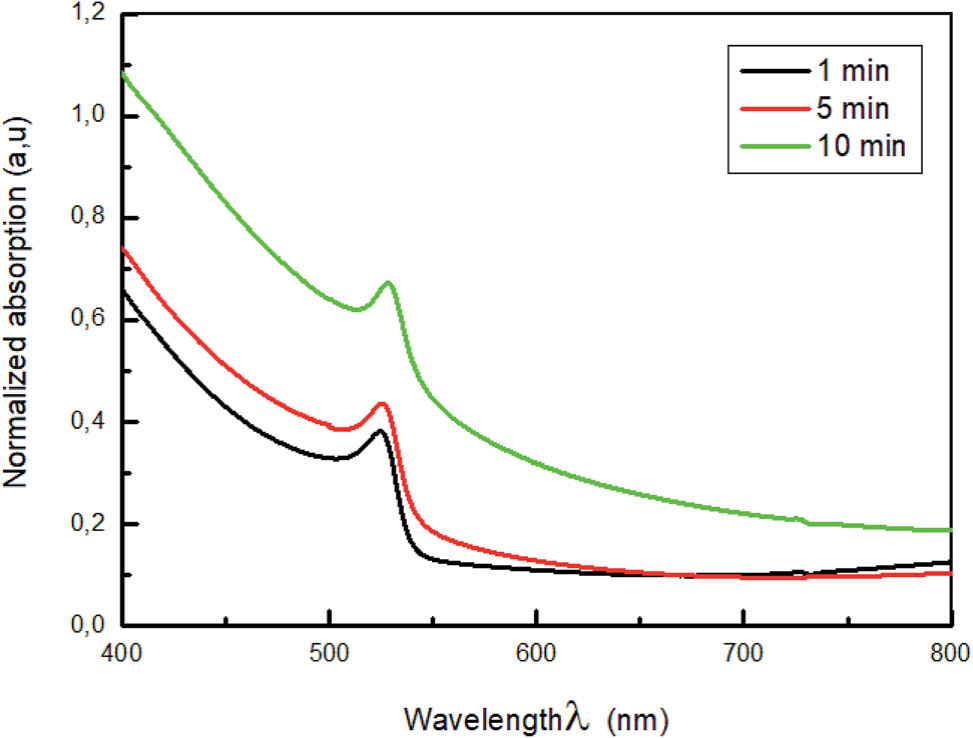
Absorption spectra of MAPbBr_3_ with different annealing time (1 min; 5 min and 10 min).

The normalized absorption of MAPbBr_3_ film annealed at 100 °C for various time (1 min, 5 min and 10 min) are displayed in Fig. 7.

As shown in Fig. 7, all spectra showed a strong absorption peak located at *λ* = 528 nm corresponding to the optical band-gap of MAPbBr_3_, *E*_g_ = 2.3 eV^50,51^ with broad absorption ranging from visible to near IR region.

It is clearly observed that increasing the annealing temperature time from 1 to 10 min lead to an increase in absorbance intensity. This can confirm the total formation of the perovskite MAPbBr_3_ layer.^52^ Furthermore, the increase of the absorbance is attributed to a change in the crystallinity of the hybrid perovskite material that causes an improvement in light scattering.

**Fig. 8:**
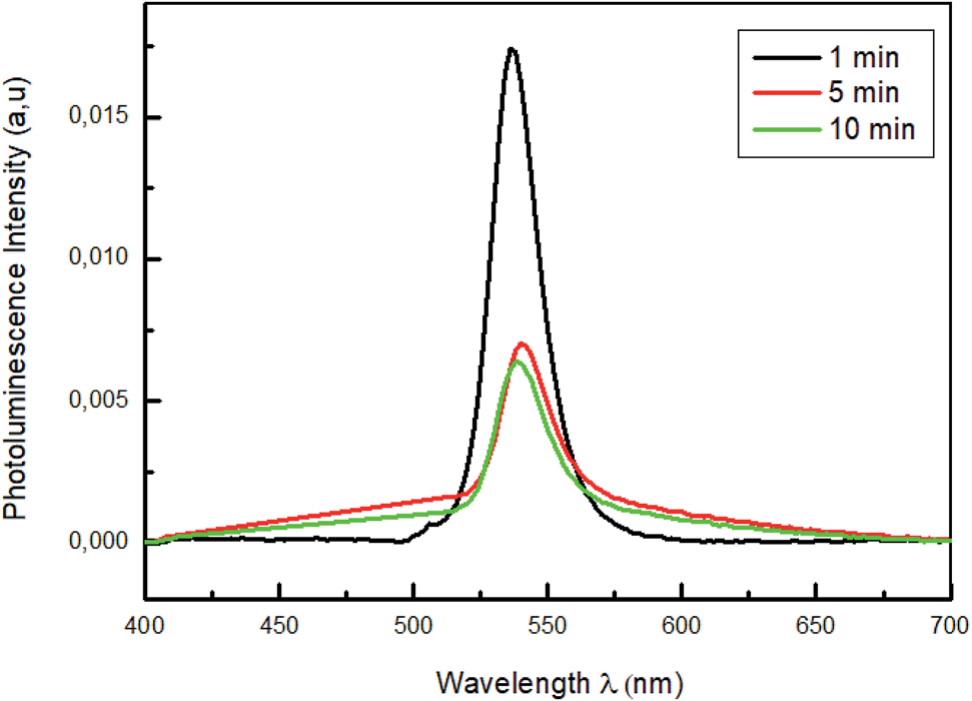
Photoluminescence spectra of MAPbBr_3_ with different annealing time (1 min; 5 min and 10 min).

Fig. 8 shows the photoluminescence spectra of PEDOT:PSS/MAPbBr_3_ films annealed for different times (1 min, 5 min and 10 min), under an excitation wavelength of *λ* = 375 nm. The PL emission characteristic peak of the MAPbBr_3_, around *λ* = 540 nm, was significantly quenched with the increase of the annealing time up to *t* = 10 min. We can notes that the increase of the annealing time had produced new dissociation interfaces and improved the exciton separation and an efficient holes transfer. Furthermore, the lowest intensity observed for the film annealed at 100 °C for 10 min is a sign of the most effective charge separation at the interface and the fast transportation of hole from perovskite films to PEDOT:PSS layers.^53^

#### 3.2.3. Photovoltaic performance

**Fig. 9:**
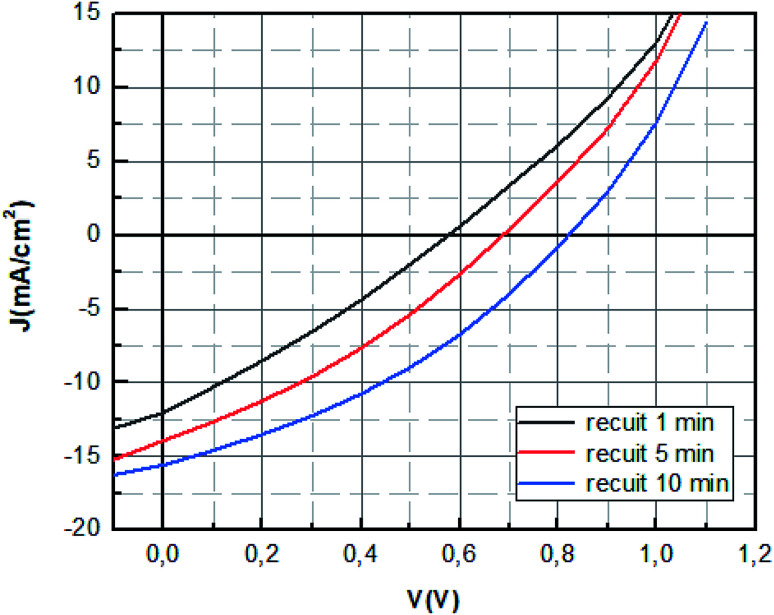
*J*–*V* characteristics of MAPbBr_3_ solar cell with different annealing time.

The characteristic curves of photocurrent density *versus* voltage (*J*–*V*) was demonstrated in Fig. 9, with the detailed *J*–*V* parameters summarized in the Table 2, where *V*_oc_ is the open circuit voltage, *J*_sc_ the short-circuit current density, FF is the fill factor, *R*_s_ is the series resistance, which indicates the total resistance of the device.

**Table 2:** Photovoltaic parameters of ITO/PEDOT:PSS/MAPbBr_3_/PCBM/Al devices with different annealing time

Annealing time (min)	*V*_oc_ (V)	*J*_sc_ (mA cm^−2^)	FF (%)	PCE (%)	*R*_s_ (ohm cm^2^)
1	0.59	12.83	30.11	2.28	15.50
5	0.69	14.46	31.55	3.14	10.11
10	0.84	16.16	35.50	4.85	6.87

It seems as listed in Table 2 that the MAPbBr_3_ layer annealed at 100 °C for 10 min showed the highest photovoltaic performance with an open-circuit voltage (*V*_oc_) of 0.84 V, a short-circuit current (*J*_sc_) of 16.16 mA cm^−2^, a fill-factor (FF) of 35.50 corresponding to a PCE of 4.85% under standard 1 sun AM 1.5 simulated solar irradiation.

The improvement of the *J*_sc_ and *V*_oc_ in the PSCs with the increase of annealing time is in correlation with the optical results obtained. It can be ascribed the better electron and holes injection in the PEDOT:PSS and PC_71_BM layers and to the better light harvest ability.

The values of *R*_s_ decreased significantly when the annealing time increased further which indicate the reduction of the losses at the interfaces sign of the good crystallization of the perovskite layer.

## 4. Conclusion

In summary, we have successfully demonstrated that high-quality organic–inorganic MAPbBr_3_ perovskite films and planar MAPbBr_3_ solar cells can be fabricated by the dip-coating method. The effects of dipping time and annealing time on the MAPbBr_3_ film quality and its photovoltaic performance were studied. The optimal deposition conditions of MAPbBr_3_ films were obtained with a 10 min dip time of the inorganic film in the organic solution, and post-annealing temperature of 100 °C for 10 min. The deposited films are well crystallized. As a result, the standard MAPbBr_3_ cell showed a power conversion efficiency of 4.85% with a *J*_sc_ of 16.16 mA cm^−2^, a *V*_oc_ of 0.84 V and FF of 35.5%. Furthermore, the dip-coating method is an effective approach for preparing high-quality MAPbBr_3_ films.

## Conflicts of interest

There are no conflicts to declare.

## Supplementary Material
